# Commentary: Characteristics of tertiary lymphoid structures in prostate cancer and the impact of neoadjuvant therapy on their formation and maturation

**DOI:** 10.3389/fimmu.2026.1925652

**Published:** 2026-08-04

**Authors:** Qingyu Wan, Wujie Chen, Jiyao Yang, Yiling Qin, Haihao Li, Ji Li, Yinglong Huang, Yidao Liu, Mingxia Ding

**Affiliations:** 1 Department of Urology, The Second Affiliated Hospital of Kunming Medical University, Kunming, Yunnan, China; 2 Department of Urology, The First People’s Hospital of Yunnan Province, Kunming, China; 3 Department of Urology, Dehong Prefecture People’s Hospital, Mangshi, Yunnan, China

**Keywords:** immunotherapy, neoadjuvant hormone therapy, prostate cancer, tertiary lymphoid structures, tumor microenvironment

## 1 Introduction

We read with great interest the article by Liu and colleagues (1), which systematically characterizes tertiary lymphoid structures (TLS) in prostate cancer (PCa) and demonstrates that neoadjuvant hormone therapy (NHT) promotes TLS formation, maturation, and immune cell infiltration. This is among the first studies to establish TLS as a favorable prognostic biomarker in PCa and, to our knowledge, the first to provide direct clinical and experimental evidence that androgen deprivation therapy (ADT) can convert prostate cancer from an immunologically “cold” tumor into a more immune-responsive “hot” tumor. We hold great admiration for the academic contributions of the research team. At the same time, we would like to offer several constructive suggestions regarding potentially extensible directions in this study, hoping to provide reference for future investigations.

## 2 Major academic contributions of this study

### 2.1 Comprehensive research framework and multi-dimensional validation system

A distinctive strength of this study lies in its establishment of a complete chain of evidence spanning from clinical pathological specimens to transcriptomics and ultimately to animal models. The authors first systematically characterized the spatial distribution, density, and maturation of TLS in three independent clinical cohorts using H&E staining and multiplex immunohistochemistry (mIHC). Subsequently, they leveraged public transcriptomic databases (2, 3) to compare TLS gene signature expression between NHT-treated and treatment-naïve patients via single-sample gene set enrichment analysis (ssGSEA) (4). Critically, paired pre- and post-NHT biopsy-prostatectomy specimens provided direct evidence of NHT-induced TLS enhancement within the same patients. Finally, the authors established an orthotopic immunocompetent mouse model using CRISPR-Cas9 gene editing of prostate organoids (TP53, PTEN, and RB1 triple knockout with MYC amplification) (5), and validated *in vivo* that ADT remodels the tumor immune microenvironment and synergizes with anti-PD-1 therapy. This research paradigm—”clinical observation → pathological quantification → transcriptomic validation → animal model verification”—not only substantially enhances the rigor and reliability of the study’s conclusions but also provides a methodological template for similar investigations of therapy-induced immune modulation.

### 2.2 TLS maturation stratification reveals differential prognostic value

The study goes beyond a simple “TLS presence or absence” dichotomy by performing a refined maturation staging of TLS—distinguishing early TLS (E-TLS), primary follicle-like TLS (PFL-TLS), and secondary follicle-like TLS (SFL-TLS), the latter characterized by CD21^+^ follicular dendritic cell networks and active germinal centers. A key finding is that only mature TLS, particularly SFL-TLS, significantly correlate with prolonged progression-free survival (PFS: P = 0.0364, HR = 0.1745), whereas immature TLS lack significant prognostic value. Similarly, higher intra-tumoral TLS density (P = 0.0016, HR = 0.079) was associated with better prognosis compared to peri-tumoral TLS. This nuanced stratification aligns with the emerging consensus in genitourinary cancers that mature TLS with functional germinal centers are critical drivers of effective anti-tumor immunity (6, 7), whereas immature TLS may fail to elicit adequate immune responses and may even be associated with an immunosuppressive phenotype (8).

### 2.3 Discovery of NHT/ADT as a TLS-inducing therapeutic strategy

This study is the first to demonstrate, in both clinical specimens and an orthotopic mouse model, that NHT promotes TLS formation and maturation. Patients in the NHT group exhibited significantly increased TLS density and maturity, along with enhanced infiltration of CD4^+^, CD8^+^, CD20^+^, and CD21^+^ immune cells. Paired biopsy analysis revealed that the detection rate of SFL-TLS increased from 0% to 65% following NHT. Transcriptomic analysis confirmed the upregulation of multiple TLS gene signatures after NHT. In animal experiments, ADT increased CD45^+^ immune cell infiltration and the proportion of CD3^+^ T cells, with a shift toward CD8^+^ predominance. The combination of anti-PD-1 and ADT exerted the most potent anti-tumor effect, with one mouse achieving complete tumor regression—an outcome not observed in any monotherapy group. This finding carries significant translational implications, suggesting that TLS induction may represent a core mechanism linking ADT to enhanced immunotherapy responsiveness in prostate cancer, corroborating the observations by Hawley et al. (9), who reported enhanced immune infiltration with anti-PD-1 plus ADT in metastatic hormone-sensitive prostate cancer.

## 3 Reflections and perspectives on this study

Notwithstanding the notable strengths outlined above, upon careful examination of the study, we believe that several aspects warrant further exploration and refinement to enhance mechanistic depth and the robustness of the conclusions.

### 3.1 The molecular mechanisms by which NHT promotes TLS formation remain to be elucidated

While the study convincingly demonstrates that NHT promotes TLS formation and maturation at the phenotypic level, the underlying molecular mechanisms remain largely unknown. Multiple potential pathways merit in-depth investigation. First, chemokine networks are critical for TLS formation—CXCL13 recruits B cells and T follicular helper cells, while CCL19 and CCL21 organize T-cell zones and promote high endothelial venule (HEV) formation (10). Does NHT upregulate these chemokines? To address this question, we propose that multiplex cytokine/chemokine profiling of pre- and post-NHT specimens be performed to identify TLS-inducing factors. Second, androgen receptor (AR) signaling in immune cells directly influences anti-tumor immunity. Guan et al. (11) demonstrated that AR activity in T cells limits checkpoint blockade efficacy, and that AR inhibition enhances CD8^+^ T-cell function and prevents exhaustion. Yang et al. (11) showed that AR regulates CD8^+^ T-cell stemness through epigenetic and transcriptional differentiation programs. Chesner et al. (12) further found that AR inhibition upregulates MHC-I expression and improves T-cell responses. However, whether these AR-mediated T-cell effects represent upstream events of TLS formation or operate through independent pathways remains unclear. Therefore, conditional knockout of AR in T cells or stromal cells could be employed to dissect cell-type-specific contributions. Third, stromal components—including fibroblasts, HEVs, and lymphatic vessels—serve as essential scaffolds for TLS formation. Does NHT modulate the fibroblast-derived lymphotoxin-β receptor (LTβR) pathway or promote vascular remodeling to facilitate TLS neogenesis (13)? Spatial transcriptomics could be utilized in future studies to map TLS-associated molecular gradients within the tumor microenvironment, thereby addressing these knowledge gaps.

### 3.2 The functional quality of NHT-induced TLS requires deeper characterization

The study primarily quantifies TLS based on morphological and immunophenotypic criteria (CD4^+^, CD8^+^, CD20^+^, and CD21^+^ staining). However, the functional quality of these TLS—that is, whether they are capable of supporting effective anti-tumor immune responses—remains incompletely characterized. Key unresolved questions include: Do NHT-induced TLS contain tumor-specific T cells? Are B cells within TLS capable of producing anti-tumor antibodies (14)? Is the functional status of CD8^+^ T cells within TLS fully ascertained—specifically, whether they are functionally active (expressing Granzyme B, Perforin, IFN-γ) or exhausted (PD-1^+^, TIM-3^+^)? While the transcriptomic analysis in the present study revealed upregulation of GZMB and PRF1 following NHT (**Figure 6C** in the original article), suggesting cytotoxic potential, direct functional validation is lacking. To further strengthen and substantiate these findings, we propose the following approaches: (A) multiplex immunohistochemistry for functional markers (Granzyme B, PD-1, FOXP3) within TLS to assess immune activation versus exhaustion; (B) isolation of TLS-infiltrating lymphocytes for T-cell receptor (TCR) sequencing to evaluate clonal expansion and tumor reactivity; and (C) assessment of B-cell maturation markers (BCL6, CD23, IgG/IgA plasma cells) to confirm germinal center functionality. These complementary strategies would provide additional validation of the current conclusions.

### 3.3 Baseline imbalance and limited sample size warrant cautious interpretation

The authors acknowledge that, in Cohort 2, patients in the NHT group had statistically higher Gleason scores than those in the non-NHT group—a baseline imbalance that may introduce selection bias. Although the paired pre- and post-NHT biopsy-to-prostatectomy design in Cohort 3 partially addresses this issue by using each patient as their own control, the sample size for the paired analysis (n = 20) remains relatively small, which may limit the generalizability of the findings. Furthermore, the mouse model utilized bicalutamide as the ADT agent, whereas clinical NHT regimens often encompass a variety of agents, including GnRH agonists, antagonists, or combined androgen blockade. Whether different ADT agents exert differential effects on TLS induction remains unclear. For future investigations, we recommend: (A) validating these findings in larger, multi-center cohorts with more balanced baseline characteristics; (B) comparing the effects of distinct ADT regimens on TLS induction; and (C) conducting prospective trials specifically designed to evaluate TLS as a secondary endpoint in NHT-treated patients.

## 4 Methodological synthesis and broader implications—paradigm reflections from this study to similar tumor marker investigations

### 4.1 The “organoid + CRISPR-Cas9 + orthotopic transplantation” model: a new gold standard for prostate cancer immunotherapy research?

Methodologically, this study elegantly demonstrates the power of integrating patient-derived or genetically engineered organoids with CRISPR-Cas9 gene editing to generate orthotopic immunocompetent tumor models. The authors created a model that recapitulates the anatomical microenvironment, genetic drivers, and intact immune system of human prostate cancer far more effectively than traditional subcutaneous xenografts (5). This represents a significant advance over immunodeficient mouse models, which are unable to support the evaluation of T-cell-dependent immune responses.

This “organoid → CRISPR editing → orthotopic transplantation” pipeline has far-reaching implications for immunotherapy research. It enables the testing of immunomodulatory agents in a genetically defined, immunocompetent environment; allows dissection of how specific oncogenic drivers—such as PTEN loss and MYC amplification—shape the immune microenvironment and TLS formation; and facilitates evaluation of combination therapies (e.g., ADT plus immune checkpoint blockade) in a clinically relevant context. However, the current model is based on normal organoids with engineered mutations rather than patient-derived tumor organoids, which may not fully capture the genomic complexity and heterogeneity of human prostate cancer.

We suggest that future studies establish patient-derived organoid (PDO) orthotopic models to preserve patient-specific genomic and immunogenic features, and employ humanized immune system mice for testing human-targeted immunotherapies, in order to explore whether these approaches influence TLS formation and immunotherapy response.

### 4.2 TLS as a “bridge” between endocrine therapy and immunotherapy—implications for combination treatment strategies

The core translational insight of this study is that NHT enhances TLS formation and maturation, thereby converting prostate cancer from a “cold” to a “hot” tumor, which in turn creates a window of opportunity for immune checkpoint blockade. This finding aligns with a growing body of evidence positioning TLS as predictive biomarkers for immunotherapy response across multiple cancer types. In melanoma, renal cell carcinoma, and non-small cell lung cancer, the presence of TLS correlates with prolonged survival and better responses to anti-PD-1/PD-L1 therapy (15, 16). The present study extends this paradigm to prostate cancer, suggesting that TLS induction may serve as a mechanistic mediator linking ADT to improved immunotherapy outcomes—a finding with substantial clinical implications. Current immune checkpoint inhibitors show limited efficacy in prostate cancer, largely attributable to its immunosuppressive tumor microenvironment and low tumor mutational burden (17, 18). By demonstrating that ADT primes the tumor microenvironment through TLS formation, this study provides a rational basis for sequencing or combining ADT with immunotherapy in clinical trials. The complete tumor regression observed in combination-treated mice underscores the potential of this strategy, echoing the enhanced immune infiltration reported by Hawley et al. (9) in metastatic hormone-sensitive prostate cancer patients receiving anti-PD-1 plus ADT. However, several critical questions remain to be addressed: What is the optimal timing and duration of ADT for TLS induction? Does TLS formation in primary tumors also occur at metastatic sites? Future studies could be designed with TLS as a biomarker endpoint to determine the optimal sequencing of ADT and immunotherapy.

### 4.3 From “TLS as a biomarker” to “TLS as a therapeutic target”—the emerging field of TLS engineering

The present study primarily positions TLS as a prognostic biomarker and a mechanistic mediator of ADT-induced immune activation. However, an emerging paradigm views TLS not merely as passive indicators but as active therapeutic targets—that is, strategies to therapeutically induce or enhance TLS to overcome immunotherapy resistance (19). In breast cancer, neoadjuvant chemotherapy has been shown to induce TLS formation, correlating with pathological complete response (20). In small cell lung cancer, LTβR agonists combined with anti-PD-L1 have been demonstrated to enhance TLS formation and anti-tumor immunity (13). In genitourinary cancers, TLS are increasingly recognized as potential targets for immune modulation (6). The present study provides proof-of-concept that endocrine therapy can serve as a TLS-inducing agent. For future investigations, we propose systematically screening for agents that promote TLS formation in prostate cancer organoid-mouse models; evaluating combination strategies (NHT plus ADT plus anti-PD-1) for maximal TLS induction and tumor control; and developing non-invasive imaging or liquid biopsy approaches to monitor TLS dynamics during therapy.

### 4.4 Expanding the horizon: TLS in other genitourinary malignancies

Although this commentary primarily focuses on TLS in prostate cancer, interpreting these findings within the broader context of other genitourinary (GU) malignancies offers equally important insights. The prognostic and therapeutic significance of TLS is increasingly recognized across the GU tumor spectrum. In renal cell carcinoma (RCC), the presence of mature TLS has been consistently associated with favorable outcomes and enhanced responsiveness to immune checkpoint inhibitors, with TLS serving as key sites for the generation of tumor-specific antibody-producing plasma cells (14). Furthermore, TLS density and maturation status have been shown to correlate with the efficacy of anti-PD-1/PD-L1 therapy in clear cell RCC, suggesting their potential as predictive biomarkers (21). In bladder cancer, although the evidence is more nascent, recent studies have begun to unravel the prognostic role of TLS, with some indicating that their presence correlates with prolonged survival and a more inflamed tumor microenvironment (22, 23). Moreover, even in rare GU tumors such as penile carcinoma, preliminary data suggest that TLS may exert a protective role, opening avenues for immune-based interventions (24, 25). Collectively, these observations across the GU malignancy spectrum reinforce the paradigm that TLS represent a shared mechanism of anti-tumor immunity, and that strategies to induce or augment TLS formation—whether through neoadjuvant hormone therapy, chemotherapy, or targeted agents—hold promise across multiple cancer types. This broader perspective underscores the generalizability of the findings by Liu et al. and provides a compelling rationale for future cross-tumor comparative studies to delineate TLS biology in a tissue- and therapy-specific manner.

## 5 Conclusion

In summary, the study by Liu and colleagues is the first to systematically characterize TLS in prostate cancer and to demonstrate that NHT promotes TLS formation, maturation, and immune cell infiltration, thereby converting prostate cancer from an immunologically “cold” tumor into a “hot” tumor. The scientific rigor of this study is particularly evident in four aspects: multi-cohort clinical validation ensures the breadth and reproducibility of the results; the paired biopsy-prostatectomy design provides direct evidence for NHT-induced TLS enhancement; the CRISPR-Cas9 organoid-derived orthotopic mouse model enables mechanistic investigation in an immunocompetent setting; and the combination therapy experiment (ADT plus anti-PD-1) provides compelling proof-of-concept for clinical translation (1). We firmly believe that this study not only establishes TLS as a favorable prognostic biomarker in prostate cancer but also provides important preclinical evidence supporting the integration of endocrine therapy with immunotherapy—with TLS induction as a central mechanism.

We sincerely hope that the suggestions proposed herein—elucidating the molecular pathways by which NHT promotes TLS formation, characterizing the functional quality of NHT-induced TLS, addressing baseline imbalances through larger-scale cohorts, and extending the organoid-CRISPR model to patient-derived systems—may serve as a source of inspiration for the authors and fellow researchers in the field. We once again thank the team led by Liu and Dong for their important academic contribution to this research area.
